# Supplementary material for: Preventing ovariectomy-induced weight gain decreases tumor burden in rodent models of obesity and postmenopausal breast cancer
Source: Breast Cancer Res. 2022 Jun 20;24:42. doi: 10.1186/s13058-022-01535-x (PMC9208221; doi:10.1186/s13058-022-01535-x)
Supplement: Supplementary file 1 — Additional file 1. Supplemental Tables 1–4. [file 13058_2022_1535_MOESM1_ESM.pdf]

Supplemental Table 1: Body weight and % body fat preOVX.

Timepoint	L[1]	OB[1]	p-value[2]
BW 9 wks	203.9 (5.2)	226.1 (3.0)	<0.001
BW 14 wks	243.8 (7.0)	277.6 (4.4)	<0.001
BW OVX	273.1 (7.2)	353.6 (9.4)	<0.001
% Fat 9 wks	12.1 (0.5)	14.0 (0.5)	0.012
% Fat 14 wks	14.9 (0.6)	19.5 (0.8)	<0.001
% Fat OVX	17.3 (0.4)	27.6 (0.9)	<0.001

[1] Mean (std.error)
[2] One-way ANOVA

Supplemental Table 2: Cumulative weight gain weeks 0 - 4 postOVX

	L		OB		Two-way ANOVA p-values		
Characteristic	AdLib[1]	WM[1]	AdLib[1]	WM[1]	Adipo	Rx	Adipo * Rx
Wt gain week 0-1	8.8 (1.6)	-0.4 (1.6)	7.6 (2.4)	-0.5 (2.5)	0.6	0.006	0.8
Wt gain week 0-2	28.0 (1.8)	1.7 (1.4)	24.1 (4.0)	-0.3 (2.9)	0.3	<0.001	0.8
Wt gain week 0-3	41.8 (3.0)	0.7 (1.5)	38.6 (4.9)	1.7 (2.6)	0.5	<0.001	0.6
Wt gain week 0-4	50.9 (4.0)	1.1 (1.2)	50.1 (5.4)	1.3 (2.1)	0.9	<0.001	>0.9

[1] Mean (SE)

Supplemental Table 3: End of Study Plasma Metabolites

	L		OB		Two-way ANOVA p-values		
Characteristic	AdLib[1]	WM[1]	AdLib[1]	WM[1]	Adipo	Rx	Adipo * Rx
Cholesterol, mM	1.7 (0.1)	1.2 (0.2)	2.3 (0.2)	1.8 (0.2)	0.008	0.010	>0.9
Triglycerides, mM	0.4 (0.0)	0.4 (0.0)	0.6 (0.0)	0.7 (0.2)	0.012	0.6	0.2
NEFA, uM	612.3 (43.4)	711.7 (75.6)	757.9 (50.3)	763.7 (72.8)	0.048	0.4	0.4

[1] Mean (SE)

Supplemental Table 4: End of Study Plasma Cytokines Altered by Weight Maintenance

Relative Levels of Plasma Cytokines Altered with Weight Mainenance		
**Cytokine**	**AdLib**	**WM**
ACTH	209.9 (20.6)	109.3 (23.1)
Adiponectin.Acrp30	42,893.2 (1,818.0)	39,426.6 (1,747.8)
AMPKalpha1	81.4 (12.2)	22.8 (7.8)
BDNF	88.9 (62.6)	19.9 (10.6)
CINC2alpha.beta	13,810.8 (1,270.4)	21,657.6 (1,906.9)
CNTF	429.0 (92.1)	239.9 (37.3)
CSK	110.6 (16.8)	51.0 (11.5)
EGFR	8.4(2.1)	1.9 (0.5)
ESelectin	721.4 (90.9)	479.9 (53.8)
IL13	403.0 (56.6)	134.7 (23.9)
IL1R6.IL1Rrp2	1,676.0 (662.9)	1,351.6 (141.6)
IL2	2,384.1 (381.7)	4,212.5 (679.5)
Insulin	197.5 (43.0)	122.2 (33.1)
Leptin(OB)	210.2 (94.0)	74.3 (21.4)
MIP1alpha	1,605.2 (183.1)	2,358.3 (235.6)
MIP2	436.8 (55.1)	532.0 (55.0)
NGFR	97.1 (14.1)	36.8 (10.2)
Osteopontin.SPP1	70.7 (13.8)	26.3 (7.1)
Thrombospondin	373.9 (43.8)	361.0 (141.4)
TIE2	182.9 (21.6)	93.8 (19.9)
TIMP2	273.8 (43.8)	119.9 (28.5)
TNFalpha	144.1 (30.7)	108.4 (38.3)
TRAIL	20,125.4 (3,109.3)	13,929.9 (3,723.6)
Ubiquitin	200.1 (17.4)	111.2 (22.9)
